# Epiviz Web Components: reusable and extensible component library to visualize functional genomic datasets

**DOI:** 10.12688/f1000research.15433.1

**Published:** 2018-07-17

**Authors:** Jayaram Kancherla, Alexander Zhang, Brian Gottfried, Hector Corrada Bravo

**Affiliations:** 1 Center for Bioinformatics and Computational Biology, University of Maryland, College Park, College Park, Maryland, 20742, USA; 2 University of Maryland Institute of Advanced Computer Studies, University of Maryland, College Park, College Park, Maryland, 20742, USA; 3 Department of Computer Science, University of Maryland, College Park, College Park, Maryland, 20742, USA

**Keywords:** genomics, visualization, epigenetics, bioinformatics, web components

## Abstract

Interactive and integrative data visualization tools and libraries are integral to exploration and analysis of genomic data. Web based genome browsers allow integrative data exploration of a large number of data sets for a specific region in the genome. Currently available web-based genome browsers are developed for specific use cases and datasets, therefore integration and extensibility of the visualizations and the underlying libraries from these tools is a challenging task. Genomic data visualization and software libraries that enable bioinformatic researchers and developers to implement customized genomic data viewers and data analyses for their application are much needed.

Using recent advances in core web platform APIs and technologies including Web Components, we developed the Epiviz Component Library, a reusable and extensible data visualization library and application framework for genomic data. Epiviz Components can be integrated with most JavaScript libraries and frameworks designed for HTML. To demonstrate the ease of integration with other frameworks, we developed an R/Bioconductor *epivizrChart* package, that provides interactive, shareable and reproducible visualizations of genomic data objects in R, Shiny and also create standalone HTML documents. The component library is modular by design, reusable and natively extensible and therefore simplifies the process of managing and developing bioinformatic applications.

## Introduction

The complex and diverse genomic data sets require flexible software libraries and tools to perform integrative data exploration and analyses. Web-based genome browsers and genomic data visualization tools like the UCSC Genome Browser ^1^ and the Integrated Genomics Viewer ^2^ are developed for specific use cases i.e., integrative data exploration of a large number of datasets for a region in the genome. Genomic exploration of data on these platforms is usually track-based, where the data is aligned to a reference genome and visualized as a line track. Since these tools are developed for specific use cases, integration and extensibility of these visualizations and libraries is a challenging task.

The Web as a platform has been used to serve static HTML documents traditionally. The implementation of HTML5 and the newer APIs made the Web more of a platform that supports rich and dynamic web applications. But HTML is still restrictive and limited to the tags/elements defined as part of the markup language and is not extensible. Various existing frameworks like Vue.js and React have introduced modular components, but components built for one framework do not work with another framework. Newer web platform APIs and technologies like Web Components introduced a standards-based component model that allows developers to create custom HTML elements that are natively extensible and reusable. Custom components work across modern web browsers and can be used along with most JavaScript libraries or frameworks designed for HTML. Web Components provide the ability to natively extend, import and encapsulate HTML elements. This makes the process of creating and managing web applications easier and a much smoother process. These components are modular, making the code cleaner and less expensive to maintain compared to JavaScript libraries and frameworks like BioJs ^3^.

**Figure 1.**
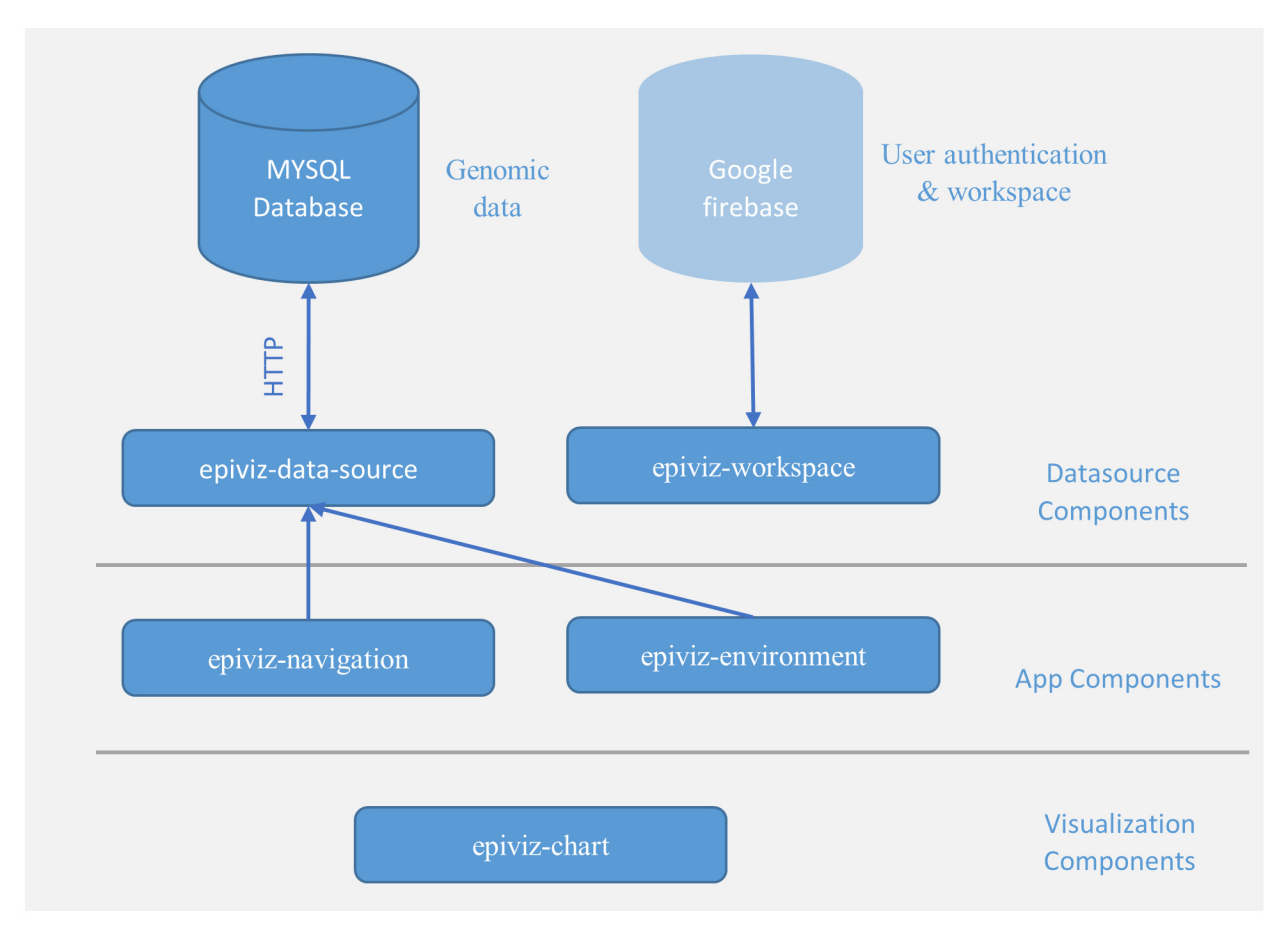
Overview of Epiviz web components architecture. The epiviz web components architecture is organized into three categories: 1) *visualization components* is a library of extensible and interactive D3JS based chart components specifically designed for genomic data; 2) *app components* are responsible for managing the layouts, events arising from genomic coordinate navigation, linking data across visualization components to implement brushing and coordinating data requests across multiple charts; 3) *datasource components* manage requests to web server or WebSocket connections using the *epiviz-data-source* component. *epiviz-workspace* handles user authentication and saves the state of the app to a google firebase instance allowing users to create shareable and reproducible visual analytics workspaces.

We present the Epiviz Component Library, an open source reusable and extensible data visualization library and application framework for functional genomic data. Building upon the Web Component framework, we developed various HTML elements/tags as part of our design as shown in Figure 1. The *visualization components* ( ***epiviz-charts)*** are the core of the library and render extensible and interactive track and feature-based charts. In addition to the chart library, we developed components for creating interactive genomic applications for different use cases and datasets. These include *app components* ( ***epiviz-navigation*** and ***epiviz-environment***) to coordinate interactions (linking data across visualizations to implement brushing and events) and manage layouts, *datasource components* including ***epiviz-data-source*** to manage data requests from a web server or WebSocket backend and ***epiviz-workspace*** for handling user authentication and to create shareable and reproducible visual analytical workspaces. The design of the component library is based on visualizations and features of the Epiviz ^4^ web application for visual exploration and analysis of functional genomic data.

Bioconductor ^5^ is an open source community that develops bioinformatics software tools and pipelines. Ease of developing integrative analyses and a framework for interactive visualizations is one of the core infrastructure needs of the Bioconductor community. Since the web components introduced in this paper can be easily embedded or integrated with any web-based application, the library reduces the effort to visualize and create applications for genomic datasets encapsulated in Bioconductor infrastructure data representations. We developed an R/Bioconductor package, ***epivizrChart*** ^6^ to visualize genomic data objects within HTML documents created using RMarkdown. We also integrated our components with Shiny ^7^, a web application framework for R to interactively visualize functional genomic data.

## Methods

### Implementation

***Visualization components***. ***epiviz-chart*** components are a collection of reusable and extensible data visualizations specifically designed for genomic data. The library provides multiple data visualizations for both location (visualizing data along the genome genes tracks ( *epiviz-genes-track*) or line tracks ( *epiviz-line-track*)) and feature based data (visualizing quantitative measurements like gene expression with scatterplots ( *epiviz-scatter-plot*) and heatmaps ( *epiviz-heatmap-plot*)). We use D3.js ^8^ (version: 3.5.17) JavaScript library to render customizable and interactive charts.

**Figure 2.**
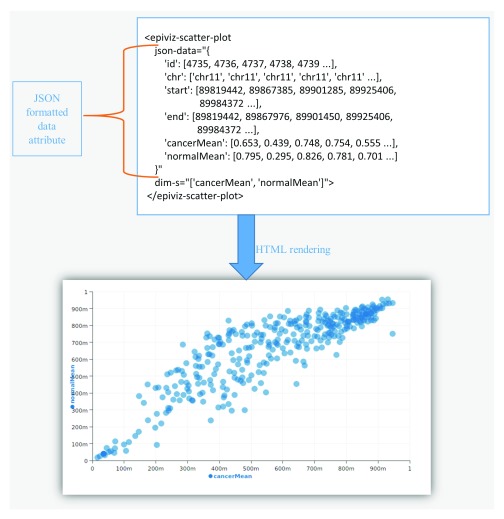
Example using components in HTML page. Epiviz components can be inserted in any HTML page using tags defined by the component library (e.g., *epiviz-json-scatter-plot* in this example). Data is supplied to the chart via the *json-data* attribute of the HTML tag. In this example, we show a sample JSON object representing genomic data. In this figure, we are only showing the first 5 data points although the plot renders more visual objects. When used in conjunction with *epiviz-data-source* components, data can be queried from a web server or via a WebSocket connection through a corresponding assignment of the *json-data* attribute. Adding the epiviz element to the HTML page renders the interactive scatter plot.

An ***epiviz-chart*** component requires two attributes to render a visualization on the page 1) *data* attribute - a JSON (JavaScript Object Notation) representation of genomic data. 2) *dimensions* (or columns) from the *data* attribute to visualize. Figure 2 demonstrates the ease of embedding or adding an ***epiviz-chart*** to a HTML document or web application.

***epiviz-chart*** components are reactive components that render the visualization only after the *json-data* attribute is initialized on the element. Any change to the *json-data* attribute triggers an event to revisualize the chart. Visualizations are extensible and easily customizable to define various settings and colors. To demonstrate the extensibility of the components, we created a component *epiviz-genes-table* extending *epiviz-genes-track* and displays a table of all the genes in the current genomic region ( Supplementary File 1).

In addition to visualizing data, chart elements can also perform client-side operations on data sets/measurements. For example, if an *epiviz-line-track* is visualizing methylation data from multiple samples (tumor and normal), samples can be aggregated using a metric (mean, min, max, etc.) to visualize the difference in methylation between normal and tumor samples. Similarly, *epiviz-heatmap-plot* interactively and dynamically clusters data and renders the clustered dendrogram. Settings are available to change clustering type and the distance metrics.

Chart components provides performance optimizations for visualizing large amounts of data by precomputing and grouping overlapping data points to a single visual object on the chart. This minimizes the number of overlapping data points to visualize and reduce rendering time of charts.

**Data model**

The *json-data* attribute on an epiviz element is a JSON object that represents genomic data in a columnar format as shown in Figure 2. The required *keys* in the JSON are *chr, start*, *end* and *data* columns to visualize. Developers can also extend the *epiviz-data-behavior* element to implement custom data parsers and formats.

**Linked data selection/brushing**

Chart components implement a linked data selection/highlighting (brushing) feature, to provide a quick overview and visually link the highlighted genomic region across all visualizations and datasets. The linking happens on the client side by finding positions that overlap with the highlighted region. In feature-based visualizations, for example in scatter plots and heatmaps, the visual objects on the chart are aggregated and mapped to multiple data objects across genomic regions. This mapping allows for implementing brushing and propagating events to other charts when using plots. In track-based visualization, events for brushing and selection are propagated based on the region ( *chr, start* and *end*) in the chart.

Another essential part of the epiviz design is that data and plots are separated. Users can visualize multiple charts from the same data object without having to replicate the data. This way data queries are made by the data object and not per chart, which leads to a more responsive design of the system.

***epiviz-chart*** components are simple user interface (UI) elements. They cannot make data requests or can directly interact with other epiviz-* elements on the page. Chart elements create hover events that propagate up the document object model (DOM) hierarchy. To build interactive web applications or to coordinate interactions by linking data across charts, implement brushing and manage data requests across chart elements, we encapsulate charts inside app components.

***App components***. ***epiviz-app*** components are abstract components that 1) Manage layouts of multiple visualizations, 2) Coordinate interactions across charts by genomic position to implement brushing, and 3) Manage data requests.

There are two different types of ***epiviz-app*** elements -

***epiviz-environment*** elements are not linked to a specific genomic region. If a genomic region ( *chr*, *start* & *end* attributes) is not initialized on the element, charts visualize the entire data set genome-wide. This helps identify patterns or interesting regions in the dataset and then investigate specific regions of interest.

**Figure 3.**
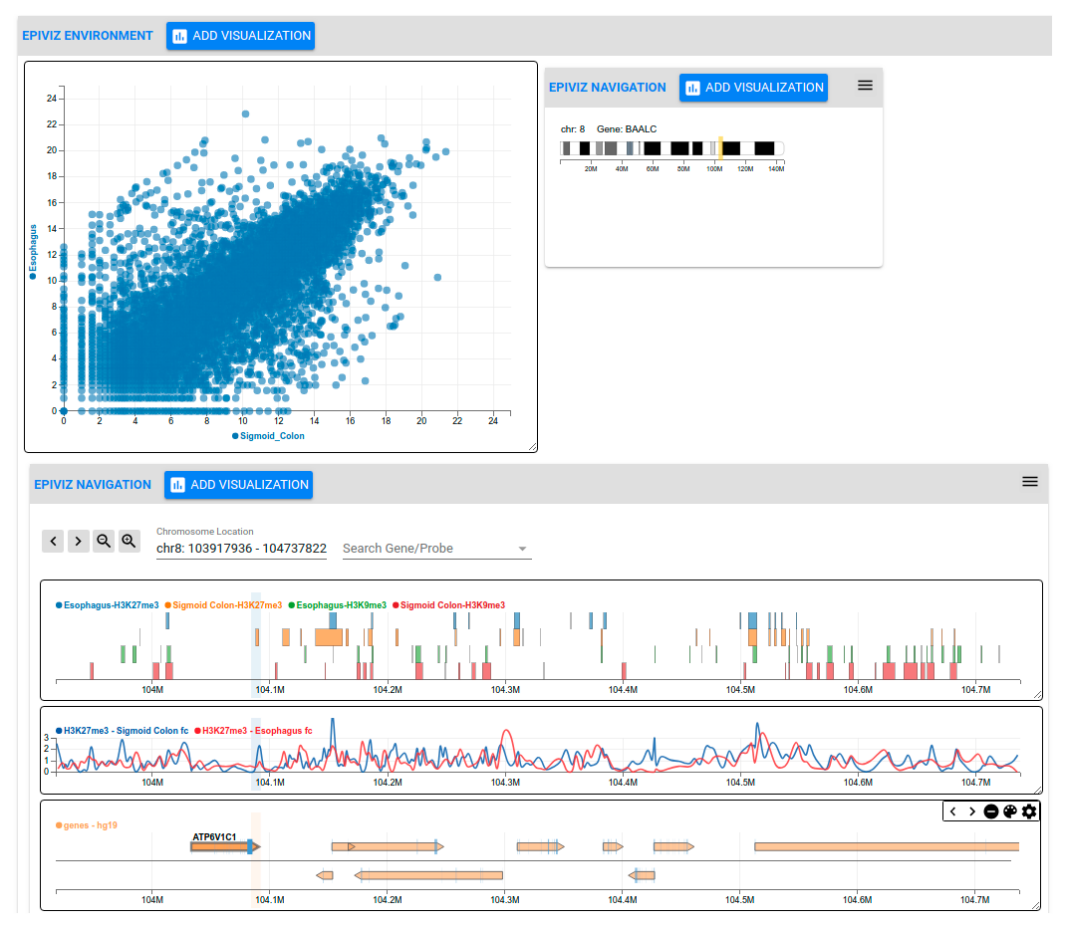
Overview of the Epiviz2 web application for Epigenome Roadmap data. In this workspace, we explore data from the Epigenome Roadmap project in two genomic regions simultaneously ( *Epiviz Navigation* components) along with a genome-wide scatterplot of gene expression (top left). The *environment* element is not constrained to a specific genomic region, and hence charts included within them visualize entire datasets. In this example, the scatter plot in the top left shows RNA-seq data for esophagus and colon tissues across the entire genome. *EpivizNavigation* components, on the other hand, are constrained to specific genomic regions. Given genomic regions or genes of interest in the dataset to further investigate, multiple navigation elements, each corresponding to distinct genomic regions can be added to the workspace. In this example, the navigation element at the bottom of the page visualizes (in order from top to bottom): 1) a genes track showing gene location span and strand, 2) a stacked-blocks track of ChIP-seq peaks in esophagus and colon across two different histone markers (H3K27me3 and H3k9me3), and 3) a line track that visualizes the fold change signal data for the same ChIP-seq data. The line track shows that the region around the gene “ATP6V1C1” shows a peak for H3K27me3 in ***Esophagus*** but not in ***Colon***. The stacked blocks track compares the peak regions with other histone markers (H3K9me3). We can also investigate this region further by exploring methylation and gene expression data from these tissues by adding a navigation element (top right). The component library provides and interactive and integrative environment for genomic data exploration. This example workspace can be accessed at http://epiviz2.cbcb.umd.edu/#/epiviz-C7O4UmIb.

***epiviz-navigation*** is a specific instance of ***epiviz-environment*** with genomic region linked to the element using the *chr*, *start* and *end* attributes. Navigation elements provide UI functionality to search for a gene/microarray probe (since we serve data from the Gene Expression Barcode project ^9^) or update the location to a specific region of interest. Figure 3 (bottom) shows a navigation element with various charts when expanded. The top header bar contains functionality to navigate left/right and zoom in/out around the current genomic location. Navigation elements implement the usual genome browser interactions (pan, zoom, location input and gene name search). The chromosome location text box identifies the current location of the navigation element and can be updated to change the genomic region. Hovering over the chromosome location sends a brushing event to highlight this region across other charts encapsulated within the component. Navigation elements can be collapsed (as shown in the top panel) to allow users to flexibly focus on specific genomic regions of interest while providing an overview of other regions of interest. When collapsed, navigation components show an ideogram of the corresponding chromosome with an indication of the specific genomic region encompassed within the components (yellow rectangle). No data requests are made from charts within collapsed navigation components.

App elements coordinate events across charts, i.e., when a chart element is highlighted, an event is propagated to all other charts in the workspace (including those visualizing genome-wide data). App elements also manage layouts for positioning and resizing chart elements. The default grid layout splits the available width into six equal columns. When charts are added to a workspace, track-based charts extend across all the six columns but plot-based chart elements only span across two columns. App components have the functionality to navigate the genome and add new visualizations. Adding a new visualization opens a measurement browser, a UI interface that allows filtering and selection of measurements across different data sets.

App components can also detect if the application or page has an active web server or WebSocket connection initialized using the datasource components. If the page has no active datasource component, interactive features that generate data requests (for example – navigating to a new genomic region or adding new charts) are disabled.

***Datasource components***. ***epiviz-data-source*** component provides functionality for the epiviz app components to interact with an active web server or a WebSocket connection. Datasource components require the API endpoint ( *provider-url*) attribute where the web server or WebSocket is located and the *provider-type* attribute that specifies if it’s a web server or a WebSocket connection. When the user interacts with epiviz components, for example, adding a new visualization or navigating to a new genomic region, these interactions generate data requests that are eventually propagated and managed by the datasource elements.

**WebServer data provider**

We developed a Python Flask (version 0.12.4) based data provider that queries genomic data stored in MySQL database and responds to data requests. The data provider enables summarization where we bin small regions together and average the value for the measurements. We see a significant improvement in draw times of charts by summarizing data as discussed in the Benchmarks section of this paper. We also implemented data import functions for commonly used Bioconductor datatypes like *GenomicRanges*, *SummarizedExperiment,* etc., in our R/Bioconductor ***epivizrData*** package.

**WebSocket data provider**

The JavaScript data types that manage genomic data in epiviz components are designed similarly to Bioconductor data types. This enables easy integration and visualization of Bioconductor data objects using the visualization components. The R/Bioconductor ***epivizrChart*** package is an API to interactively visualize Bioconductor data objects. We discuss more about ***epivizrChart*** package in the Use case section of the paper.

***Workspace component***. ***epiviz-workspace*** component is built upon the Google Firebase infrastructure to manage user authentication, create shareable and reproducible visual analysis workspaces. Workspace components are easily reconfigurable and allow developers to customize this component to their firebase instance.

### Operation

Web components are a set of standardized browser APIs still being implemented across various browsers. Web components implement the Shadow DOM feature, wherein the element defined by the component is rendered separately from the rest of the HTML document avoiding namespace collisions and is isolated to keep element styling and access private to the element. Web Components are natively supported in Chrome and Safari and are still in development in Mozilla Firefox and Microsoft Edge browser.

Epiviz Components are developed using the Google Polymer library. For browsers without native web component support, the Google Polymer library provides polyfill that helps developers use components seamlessly with little performance overhead. It uses a dynamic loader to lazy load polyfill libraries for missing implementations. Documentation on attributes and methods in epiviz components is available from GitHub. The component library can visualize data by adding the chart tag to a HTML page with the data attribute as shown in Figure 2.

The epivizrChart package requires R version 3.4.0 or higher and packages from Bioconductor version 3.6 or higher. The memory requirements for using the epivizrChart package depends on the size of the dataset. However, for most use cases, a standard laptop will handle most applications visualizing data using the component library and the epivizrChart package. To visualize a Bioconductor data object, supply the supported object to the *epivizChart()* function.

## Use cases

### Epiviz2 web application

***Epiviz2*** is an interactive and integrative genome browser that sends requests to a Python Flask data provider and a MySQL database. ***Epiviz2*** allows users to interactively explore and simultaneously visualize datasets across multiple genomic regions, a feature not available in most current genome browsers. The real advantage of the genome browser lies in the ability to visualize data from multiple regions of the genome or the entire dataset to identify genomic regions of interesting patterns or outliers. Users can then further explore and visualize annotations or measurements from other datasets in these regions to gain insights. Figure 3 illustrates this workflow of exploratory data analysis. The gene expression scatter plot is encapsulated inside the environment element and visualizes the entire dataset, whereas the navigation elements are linked to a specific genomic region. We also implemented a color by region for genome-wide scatter-plots, where visual objects in the scatter plot will be colored with a different color specific to each of the genomic regions shown in navigation elements. Our instance of the ***Epiviz2*** application is hosted here.

The ***Epiviz2*** instance we host at the University of Maryland contains data from the NIH Roadmap Epigenomics ^10^ project. The NIH Roadmap Epigenomics Mapping Consortium leverages next-generation sequencing technologies to map DNA methylation, histone modifications, chromatin accessibility and small RNA transcripts in tissues selected to represent the normal counterparts of tissues and organ systems frequently involved in human disease. Our instance of the roadmap database contains DNA methylation, RNA seq, and histone modification (for markers: h3k9ac, h3k9me3, h3k27ac, h3k27me3) fold change and peak data for seven different tissue types – Breast Myoepithelial cells, Brain Hippocampus Middle, Lung, Liver, Sigmoid Colon, Pancreas and Esophagus. The corresponding data files are downloaded from Bioconductor’s ***AnnotationHub*** repository and imported into the MySQL database using the functions available in the ***epivizrData*** package.

### epivizrChart R/Bioconductor package

**Figure 4.**
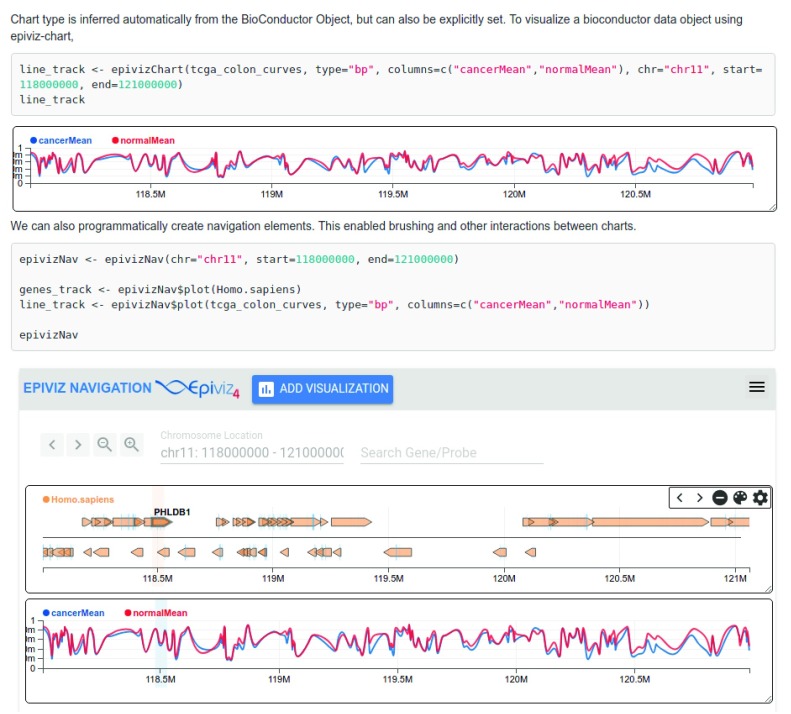
Interactive visualization of R/Bioconductor data objects using the *epivizrChart* package. This figure is part of an RMarkdown document and demonstrates the ease of integrating the visualizations from the Epiviz component library with existing frameworks. The *epivizChart* function infers the chart type based on the data object parameter. *“Homo.sapiens”* from the top panel is a UCSC Gene annotation object for human hg19 reference genome and is visualized by *epivizChart* as a genes track. t *cga_colon_curves* is a sample dataset from The Cancer Genome Atlas for colon tissue. This is a *GRanges* object and is visualized as a Line Track. The *epivizrChart* package can also programmatically create navigation elements. This enables interactions and brushing across the charts as shown in the bottom panel around the gene “PHLDB1”. A vignette describing more examples and use cases is available in the package either on the GitHub repository or through Bioconductor ( http://bioconductor.org/packages/release/bioc/html/epivizrChart.html).

The Bioconductor open source software community creates bioinformatics workflows and pipelines to analyze and visualize genomic data sets. To support interactive visualization of Bioconductor data objects, we developed an R/Bioconductor package ***epivizrChart***, an API package to programmatically create and visualize genomic datasets using epiviz components without having to import data into a MySQL database. ***epivizrChart*** demonstrates the ease of integration with existing frameworks and can create interactive web pages or RMarkdown documents as shown in Figure 4. Integrating with a statistical and powerful state-of-the-art bioinformatics data analysis platform allows users to quickly explore, analyze and visualize genomic datasets with various packages available through Bioconductor.

***online vs offline***. Using the ***epivizrChart*** package in an *online* mode creates an active WebSocket server and allows interactions between the components and the R-session. In *online* mode, components make data requests using the WebSocket connection. In *offline* mode, data is attached to the components and a standalone HTML page is generated. This allows researchers to create interactive, shareable and reproducible visualization documents.

**Figure 5.**
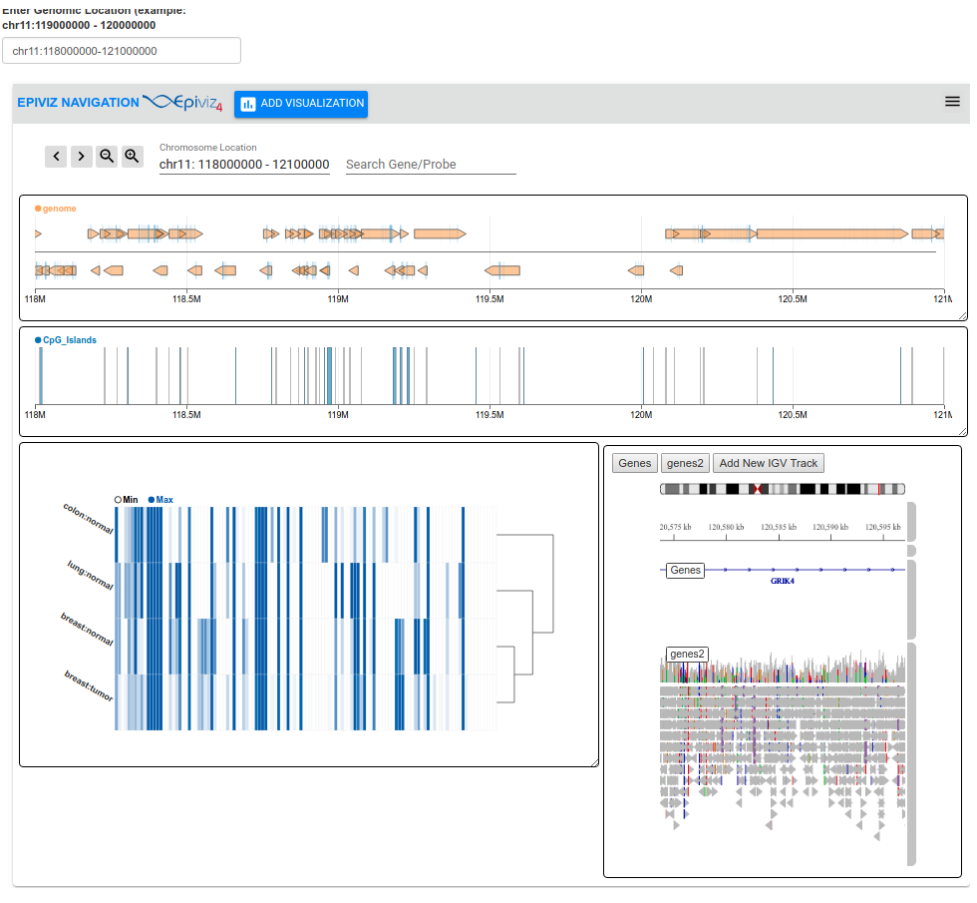
Interactive visualization of R/Bioconductor data objects in Shiny. In this Shiny application, we explore gene expression from the Gene Expression Barcode Project ^11^ for colon, lung and breast tissues for tumor and normal samples as a heatmap. We visualize annotation tracks for the genes and position of CpG islands in the current region. We also integrated IGV with epiviz components and the igv track (bottom right) displays the gene position and the aligned illumina reads for HG01879 sample from 1000 genomes ^12^ project. The IGV track queries the file directly to get data and visualize the reads. We also have a genomic location text box (top left) that is a non-epiviz component and can be used to interact with epiviz components within the Shiny application. Changing the location, updates the genomic region in the navigation element and all charts.

***Integration with Shiny***. Shiny is a web application framework to create standalone web applications on a webpage or in an RMarkdown document. Since Shiny supports HTML, epiviz components can be embedded or integrated in Shiny applications or dashboards to interactively visualize genomic data. The vignette *IntegrationWithShiny.Rmd* in the *epivizrChart* package demonstrates 1) a simple application that integrates Shiny to visualize R/Bioconductor data objects using epiviz components 2) interactions with non-epiviz components in Shiny as shown in Figure 5.

## Benchmarks

**Figure 6.**
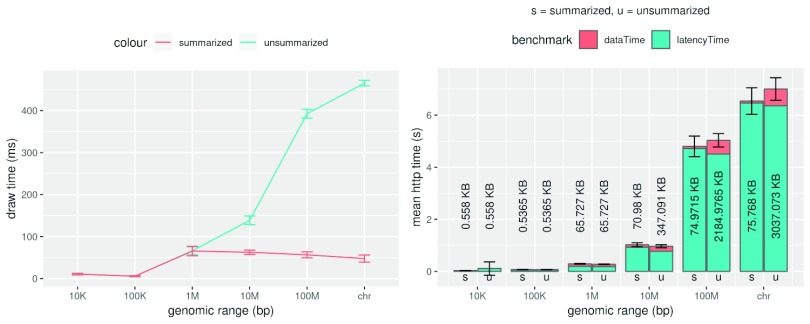
Effect of data summarization on the Epiviz Python data. Here we compare average data request and data rendering time for continuous data along the genome to study the effect of summarizing data on the data backend across 10 runs. Lines for ‘unsummarized (u)’ correspond to the previous Epiviz implementation where all data within a genomic region is returned by the php-backend to the web browser client. Lines for ‘summarized (s)’ correspond to our new implementation of the python-data backend, where data summarization within genomic regions is performed in the backend. The left panel shows the mean draw times between these scenarios where we see a significant improvement in the draw times when the data is summarized in the backend. The bar plot in the right panel shows the total http time and is separated to show mean latency times and data transfer times. The number of bytes transferred for summarized and unsummarized backends is also displayed. The error bars represent one standard deviation away from the mean draw time in the left panel and mean http time in the right panel. We observe that the total http request time (summarization plus data transfer) is comparable to transfer time for the larger unsummarized data scenarios.

We use the google chrome headless *puppeteer* tool to measure request times and chart draw times to compare our web component implementation of the ***Epiviz2*** application (with Python-MySQL backend) to the current ***Epiviz*** ^4^ application (with PHP-MySQL backend). We compare the times by varying the genomic region on the scatter plot component ( ***epiviz-scatter-plot***) across two different backend implementations: 1) Summarized responses (current implementation), where we bin the genomic region into 2000 intervals and average the data values for the measurement within each interval and, 2) Unsummarized responses (previous epiviz implementation), where the entire dataset for the region is sent back to the UI. When visualizing large genomic regions, data points tend to overlap on scatter plots and other visualizations because of pixel and chart size limitations on the page. Summarizing reduces the draw times in rendering charts because of fewer overlapping points as shown in Figure 6. However, the response times for data requests have not changed significantly because the computation time for summarization is usually similar to the time taken to transfer the entire dataset in the unscaled implementation. The scripts for the benchmarks are available in the ***epiviz-chart*** GitHub repository. The benchmark scripts can also save a screenshot of the page rendered to make sure that the page is completely loaded and rendered.

## Discussion

The component library is an extension to our ***Epiviz*** web application for visualizing functional genomic data sets. The component library is our solution to creating reusable and extensible visualization elements that work with any modern web browsers. The value of a data visualization library depends on its usability and easy integration with existing web frameworks. Epiviz components can be integrated with any framework that supports HTML.

The Web has now become the platform for application development and the demand for modular, extensible and reusable frameworks like web components is on the rise. Since epiviz components are modular, we believe it simplifies the process of developing and managing genomic web applications. We also welcome developers to contribute to and extend our component library.

## Conclusion

To our knowledge, the Epiviz component library is the first genomic data visualization library based on web components. The library provides an easy and efficient way for bioinformatics developers to add interactive data visualization features to their web applications or datasets with minimal programming experience. It is cross-platform, modular and runs on any modern web browser. We introduced our ***Epiviz2*** web application to demonstrate the features and interactions that can be developed using the component library. We also showed the ease of integration with other frameworks by the R/Bioconductor ***epivizrChart*** package, that provides interactive, reproducible visualizations of data objects in R and also create interactive standalone HTML documents.

## Future work

One of the advantages of web components is that HTML is now more readable. With a more declarative implementation, elements can be self-descriptive. We would like to implement a visualization grammar ^13^ similar to ggvis as attributes/properties on the epiviz elements. We plan to further develop the library to extend our current set of visualizations and support various genomic data types including those implemented in Metaviz ^14^ an interactive and statistical metagenomic data browser. We plan to implement canvas-based rendering of charts to scale and significantly reduce draw times especially when rendering large datasets.

## Data availability

The Datasets used for the use case describing the Epiviz Application come from the NIH Roadmap Epigenomics Project. The data files are downloaded from Bioconductor’s ***AnnotationHub*** repository and imported into the MySQL database using the functions available in the ***epivizrData*** package. For the epivizrChart package, the datasets used are included as part of the package. The vignettes describing the use cases are also available on GitHub or through Bioconductor.

## Software availability

Epiviz component library is open sourced and is available on GitHub. The collection of components discussed in this article are available at:

- epiviz charts - http://github.com/epiviz/epiviz-chart
- epiviz data - http://github.com/epiviz/epiviz-data-source
- epiviz workspace - http://github.com/epiviz/epiviz-workspace
- epiviz app - http://github.com/epiviz/epiviz-app

The scripts for benchmarks are available in the ***epiviz-charts*** repository. R/Bioconductor ***epivizrChart*** package is available either through Bioconductor ( http://bioconductor.org/packages/release/bioc/html/epivizrChart.html) or GitHub ( http://github.com/epiviz/epivizrChart). Both the respositories also contain the vignettes described in Figure 4 and Figure 5. The Python Flask API data provider is available at http://github.com/epiviz/epiviz-data-provider. Documentation is available at http://epiviz.github.io.

Archived source code at the time of publication – https://doi.org/10.5281/zenodo.1299990 ^15^

**Software license**: MIT License.

## Supplementary material

Supplementary File 1 – Document containing extending-genes-track contains code that extends an existing epiviz chart component (epiviz-genes-track) and displays a table of genes and their genomic positions.
