# Supplementary material for: Epiviz Web Components: reusable and extensible component library to visualize functional genomic datasets
Source: F1000Res. 2018 Jul 17;7:1096. [Version 1] doi: 10.12688/f1000research.15433.1 (PMC6092909; doi:10.12688/f1000research.15433.1)
Supplement: Supplementary file 1 [file f1000research-7-16818-s0000.tgz › 585eb9d1-64e8-473f-b061-242f0785caca.docx]

**Extending an epiviz genes track**

Epiviz web components are natively extensible and resuable. We demonstrate the extensibility by extending *epiviz-genes-track* to display a table with genes and their genomic positions. The code snippet loads the commonly used mixins/behaviors in epiviz components. These behaviors implement features that let developers customize new charts like settings, colors, managing events and linking data across charts (brushing). The helper function `get_json_data` returns the json data that gets rendered onto the page. The code uses a Polymer helper element *dom-repeat* that iterates through the json-data and renders a table in the DOM.

1. <link rel="import" href="../polymer/polymer-element.html">
2. <link rel="import" href="chart-behavior.html">
3. <link rel="import" href="chart-settings.html">
4. <link rel="import" href="chart-colors.html">
5. <link rel="import" href="chart-remove.html">
6. <link rel="import" href="chart-grid-behavior.html">
7. <link rel="import" href="epiviz-genes-track.html">
9. <dom-module id="epiviz-genes-table">
10. <template>
11. <div id="chart" on-drag="hostDragged" on-mouseover="hostHovered" on-mouseout="hostUnhovered">
12. <div id="{{plotId}}"></div>
13. <table class="gridtable">
14. <thead><tr><th>Gene</th><th>Start</th><th>End</th></tr></thead>
15. <tbody>
16. <template is="dom-repeat" items="[[geneTable.start]]" as="row" initial-count="30 ">
17. <tr><td>[[row.metadata.gene[index]]]</td><td>[[row.start[index]]]</td><td>[[row.end[index]]]</td></tr>
18. </template>
19. </tbody>
20. </table>
21. </div>
22. </template>
23. <script>
24. class EpivizGenesTable extends EpivizGenesTrack {
25. static get is() { return 'epiviz-genes-table'; }
26. _draw() {
27. var geneTable = this.get_json_data();
28. this.set("geneTable ", geneTable);
29. }
30. };
31. customElements.define(EpivizGenesTable.is, EpivizGenesTable);
32. </script>
33. </dom-module>
