# Supplementary material for: Epiviz Web Components: reusable and extensible component library to visualize functional genomic datasets
Source: F1000Res. 2018 Jul 17;7:1096. [Version 1] doi: 10.12688/f1000research.15433.1 (PMC6092909; doi:10.12688/f1000research.15433.1)
Supplement: Peer review history

# Peer review: Referee response for version 1

**DOI:** 10.5256/f1000research.16818.r36145

**Published:** 2018-08-14

**Authors:** Zhaohui Steve Qin

**Affiliations:** 1 Department of Biostatistics and Bioinformatics, Rollins School of Public Health, Emory University, Atlanta, GA, USA

In this paper, Kancherla and colleagues present the Epiviz Component Library, an open source reusable and extensible data visualization library and application framework for functional genomic data. According to the authors, the Epiviz component library is the first genomic data visualization library based on web components. The library provides an easy and efficient way for bioinformatics developers to add interactive data visualization features to their web applications or datasets with minimal programming experience.

 Overall, I found the tools describe in the paper very useful and promising. It makes complex visualization of multiple types of omics data easy and convenient. The new infrastructure is built upon the established epiviz tool that the group has developed earlier. This group is highly experienced in genomics data visualization and are developing state-of-art infrastructure using the latest technologies. The utilities of the tools great outweigh their limitations and shortcomings. I only have a few minor points.

 Figure 3. In this use case, I don’t quite understand the connection between the gene expression scatter plot and the other panel. For me, the figure is more like the demonstration of all types of plots it can produce. It is not obvious what biological insight can be gleaned from the plots. It is unclear what kind of biological question the user is trying to ask when making these plots. To be fair, this is always difficult. In most cases, discovery is made unintentionally, out of luck. Hence just demonstrating all the plotting capabilities is probably okay. If so, may be a systematic catalog of all the plots that can be produced will be helpful.

 Figure 6 is very informative and interesting. I noticed that the numbers of bytes transferred for summarized and unsummarized backends are exactly the same for 10K, 100K and 1M, but dramatically different for 10M, 100M and chr. Why is that?

 It would be very helpful to the general audience if the authors can articulate in more details, and intuitively, the benefits and advantages of web components-based visualization library for genomics data, compared to the current technologies.

I have read this submission. I believe that I have an appropriate level of expertise to confirm that it is of an acceptable scientific standard.

# Peer review: Referee response for version 1

**DOI:** 10.5256/f1000research.16818.r36142

**Published:** 2018-08-08

**Authors:** Ian Holmes

**Affiliations:** 1 Department of Bioengineering , University of California, Berkeley, Berkeley, CA, USA

This paper describes a library for integrative genomic data exploration, using modern web technologies, particularly HTML Web Components, with solid R integration. The interactive figures in the paper are very interesting, and show how the library can be used to build basic dashboards or RMarkdown pages with interactive charts and genome views. The dashboards look a little spartan, and a bit cryptic at times, but are a great illustration of the potential for this kind of thing and could develop into something really powerful. Plus, with the Bioconductor integration, they offer a lot of ready-to-go useful data for use cases around human genomics. All round a good contribution, although I think marred (very slightly) by some opening text in the paper which presents the software as being very general-purpose and detached from specific use cases - a style of presentation which I view as a mistake that risks detracting from a reader’s clear understanding of what the software can actually do. Modularity is a virtue, but developers often overestimate readers' level of interest in it.

 For example, according to the authors, previous genome browsers have all been based on “specific use cases”, whereas Epiviz Web Components are more amenable to “integration and extensibility”. On closer reading, it turns out that the primary use case (the NIH Roadmap Epigenomics project) is just a bit further down in the paper, as is the platform (R/Bioconductor) currently required to load any useful data or the documentation vignettes. So I think the generality of the tool is possibly a bit hyperbolically described, in a way that risks obscuring the actual current uses (and software dependencies) of the tool.

 The authors claim that, in Epiviz Web Components, they have developed the first genome browser that uses Web Components, which form a collection of browser features and APIs that have emerged from JavaScript libraries like React. Due to the fast-changing nature of the web, technologies such as this emerge with rapidity and regularity, and I find it plausible that this is the first genome browser to use these tools. They should be useful and it’s interesting to see them described in practice. The Epiviz Web Components tool is a useful addition to the bioinformatics visualization ecosystem, bridging R and the modern web.

 With that said, I think perhaps that web bioinformatics software - and its associated publications - should be measured on several axes. Compliance with emerging web standards (such as Web Components) is certainly one such axis, but compliance with established bioinformatics standards would be another axis (how many formats/database schemas does the software support? how many other resources does it integrate with? what choices were made about which data sources to support, and which to omit? are those choices explained in the text? is there a guiding philosophy that can help inform readers who might be considering using this software?)

 In terms of bioinformatics compatibility, bioconductor’s AnnotationHub seems intended to be the primary way of loading data into the browser, though it isn’t quite presented this way. Bioconductor is mentioned in the abstract as an example that was developed “to demonstrate the ease of integration with other frameworks”; I think it would be more accurate to say that Bioconductor is the only framework that this tool supports, and while the tool was written in such a way as to be hypothetically platform-independent, that hypothesis has not yet been seriously tested. 

 Epiviz Web Components does have its own JSON formats for data, which is promising in terms of backing up the claim that data import would be straightforward, but as far as I can tell there are no tools to import common file formats (FASTA, GFF, etc) and it’s not clear whether there are any plans for developing such import tools, or if the Bioconductor dependency will in practice be permanent.

 The paper’s interactive figures (Figures 3 & 4) are intriguing. I unfortunately had some problems installing the R from source (several dependencies of epivizrChart failed to build on my Mac Pro running macOS Sierra 10.12.6, R version 3.3.3), so I have not tried them on my local machine, only the JavaScript running in the web browser client. I found the captions to the Figures hard to follow: for example, I found myself wondering if there was a way for the user in the web browser to figure out how the components in Figure 3 (the genes track, stacked-blocks track, and line track) are linked, to use the same underlying data sets? In other words, does that linking only happen via configuration files and other back-end interfaces, or is it exposed to the end user? How were the markers H3K27me3 and H3k9me3 selected, why esophagus & colon, why drill into methylation and expression, what’s the biomedical back-story here? And just in terms of how to use the app to explore these cases as described, there could be more exposition. There are quite a lot of interactive buttons and menus and nested windows, so I was looking around for explanations of all that, passing by the documentation at https://epiviz.github.io/epiviz-chart/ (which is apparently oriented toward developers, not end-users, and is pretty minimal) and ending up at the https://epiviz.github.io/ video tutorial (“Epiviz quick tour”). I couldn’t find any thorough written description of the Epiviz web app, only that 3-minute YouTube video. I’m not very confident in my understanding of the totality of its capabilities after watching the video and looking at Figure 3. The potential to build pages out of RMarkdown, as shown in Figure 4, is pretty exciting.

 In summary: I would suggest improving the article by adding more extensive descriptions of the biological use cases, and how they might be investigated using the tool. Make another video if you insist, but what I’d really be looking for is clearly-written tutorial text with figures. This will complement and enhance the current concise description of the software, and offer an alternative approach for a broader class of readers.

I have read this submission. I believe that I have an appropriate level of expertise to confirm that it is of an acceptable scientific standard, however I have significant reservations, as outlined above.

# Peer review: Referee response for version 1

**DOI:** 10.5256/f1000research.16818.r36143

**Published:** 2018-08-03

**Authors:** Bianca H Habermann

**Affiliations:** 1 Aix-Marseille Univ, Aix-Marseille Univ, CNRS, IBDM UMR 7288, Marseille, France

Kancherla et al describe a set of re-usable web-components for the Epiviz tool, which are designed for visualizing genomic/epigenetic data in an integrative and interactive way. The components include the visualization components, app components and datasource components, are designed in a modular way and can be integrated with JavaScript libraries and other HTML frameworks. Moreover, there is an R-version available, which can be used with R / Shiny and to produce stand-alone HTML documents.

 The general concept and design of the Epiviz web components is very good. With growing amounts of -omics data, we need easy-to-use, re-usable and extensible code for interactive data visualization, since visualizing large-scale data enhances our understanding of inherent trends and features of the datasets under analysis.  We therefore have a growing need for easy-to-use and re-usable software for their visualization; re-useable and extensible tools like Epiviz are highly sought-after.

 The manuscript is in general well written and the software has a nice look and feel. Though, there are a couple of points that should be addressed prior to indexing.

  

- While I find the description of the tools quite good and explicit, the description of the used biological examples, as well as the plots, is in my view too sparse. In fact, visualization only makes sense to the reader, if he/she understands, what is plotted and how to interpret the plots. With the manuscript at hand, as well as the user manual, this is not made easy. For instance, there is a general lack of axis labels on the scatter plots. E.g. Figure 2 and 3 show scatter plots, however from different data sources (peak positions/height or differential detection(?) on the genome, RNA-seq data (?)). What are the numbers on Figure 3 (ie the scatter plot of the RNA-seq data)? Are these rpkms? Log2 fold change? As the plots are quite useful and might be used directly for publication, this is a feature that should be implemented.
- Also, in the same Figure/example (methylation workspace following the given link of the legend to Figure 3), how does the RNA-seq data relate to the chip-seq/methylation data? Is there a possibility to see the identity of the genes, when hovering over the dots in the scatter plot? At least for the pre-selected example shown on the web-page, there is no brushing over from the zoomed-chromosome plot to the scatter plot and vice versa. However, this feature seems to work, when changing the chromosomal region. Is this due to the fact that the genes in the selected region are not present in the RNA-seq data? If so, the authors should think about changing the selected region for their demonstration.
- Also, what is the relationship of genes in the scatter plot that are highlighted together, when one is selected? Do they share the same enhancer/methylation peaks?
- It might be useful to provide information on the genes selected in the RNA-seq plot also via an info box or pop-up, which shows the name of the gene(s) and its(their) associated differential expression values.
- Is there the possibility to e.g. change the region of the chromosome-zoom/the chromosome view, when selecting dots in the scatter plot of the RNA-seq data? This seems currently not possible, however would be desirable.
- In the same example, the brushing of the chromosome view vs the zoomed-in chart does not work. When changing the genomic region, there is no highlighting any more in the chromosome view, so only the pre-selected region seems to work. This needs to be fixed.
- Finally, in the same plot, changing the chromosome for zoomed-in visualization has no effect on the whole-chromosome view, which I assume should also be updated. In my opinion, the whole-chromosome view is in its present form not really useful and could be omitted.
- In the same workspace (methylation workspace), there is a scatter plot at the bottom of the page; what do the numbers in the scatter plot refer to? See data labels problem already discussed above.
- On the binning of data values, the authors describe that the genomic region is binned into 2000 intervals. To which overall length does this refer to? Is always a fixed length chosen or can the user determine the length? Binning e.g. 100000 bp would then give quite different results than e.g. 1000000 bp.

 

 There are also some errors in the manuscript, more specifically in the description of Figure 3. It currently states:

 “In this example, the navigation element at the bottom of the page visualizes (in order from top to bottom): 1) a genes track showing … “

 Instead, it should read, I assume, as the genes track is at the bottom of the figure:

 “In this example, the navigation element at the bottom of the page visualizes (in order from **bottom to top**): 1) a genes track showing … “

I have read this submission. I believe that I have an appropriate level of expertise to confirm that it is of an acceptable scientific standard, however I have significant reservations, as outlined above.
